# Design and fabrication of a zero-bias visible light photodetector based on a coumarin-derived Schiff base thin film

**DOI:** 10.1039/d5ra09436d

**Published:** 2026-01-26

**Authors:** Ishanki Sharma, Jagadish K. A., Suranjan Shil, Dhananjaya Kekuda, N. V. Anil Kumar

**Affiliations:** a School of Basic Science, Humanities and Management, Manipal Institute of Technology, Manipal Academy of Higher Education Manipal India nv.anil@manipal.edu; b Manipal Centre for Natural Sciences, Manipal Academy of Higher Education Manipal India

## Abstract

A self-powered visible-light photodetector was fabricated using a coumarin-derived Schiff base, 4-FMCSB, as the active layer. The compound was synthesized *via* a two-step process and characterized using FT-IR, NMR, MALDI-TOF-MS, and UV-vis spectroscopy. The optical band gap of 4-FMCSB was determined to be 2.33 eV, with DFT calculations confirming a HOMO–LUMO gap of 2.79 eV. Thin films of 4-FMCSB were deposited on ITO substrates *via* spin coating, and aluminium contacts were thermally evaporated to form Al/4-FMCSB/ITO photodetectors. The device exhibited a strong rectification ratio, rapid photo response (rise time: 510 ms; fall time: 210 ms), and efficient operation under zero bias. Key performance metrics included a responsivity of 12.6 mA W^−1^, detectivity of 9.31 × 10^10^ jones, and a linear dynamic range of 35.94 dB. These findings underscore the potential of coumarin-based Schiff bases in advancing the design of next-generation, energy-efficient optoelectronic devices.

## Abstract 2

A self-powered visible-light photodetector was fabricated using a coumarin-derived Schiff base, 4-FMCSB, as the active layer.

## Graphical abstract

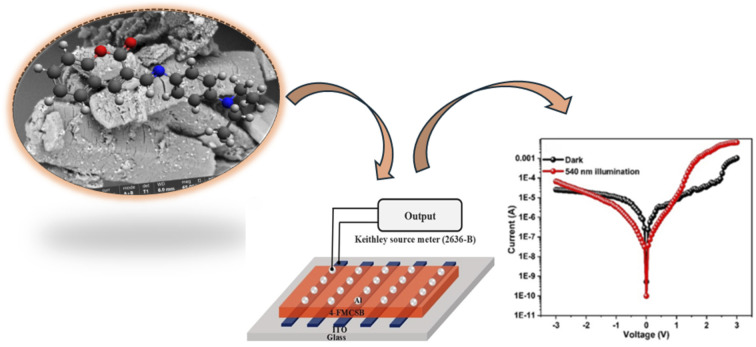

## 1 Introduction

Over the past few years, the scientific community has actively pursued the development of energy-efficient and sustainable energy detection systems in response to the rapid growth in population and the corresponding increase in energy demand.^1^ Photodetectors have attracted significant research interest due to their wide-ranging applications across various industries, including military defense, environmental monitoring, flame detection, space communication, chemical analysis, industrial quality control, and optoelectronics.^2^ Semiconductor optical properties require significant attention due to their essential applications in industrial, commercial, and military optoelectronic devices, such as light-emitting diodes (LEDs), solar cells, and photodetectors.^3^

Photodetectors are optical devices that convert incident photons into an electrical signal. The photoconductivity effect, fundamental to photodetectors (PDs), involves the generation of electron–hole pairs when radiation with energy equal to or greater than the semiconductor band gap (*E*_g_) is absorbed. These charge carriers contribute to photocurrent, driven either by an external bias (conventional PDs) or an internal built-in electric field (self-powered PDs). Conventional PDs require an external power supply, increasing circuit size and complexity. In contrast, self-powered PDs operate using internal potential differences, which enables a compact design and improved energy efficiency. Additionally, they offer advantages such as reduced power consumption and suitability for operation in harsh environments.^4^

Self-powered photodetectors (PDs) based on the photovoltaic (PV) effect in p–n junctions have garnered significant interest due to their ability to operate without external power. This feature is particularly beneficial for IoT and wearable electronics, where power availability is limited. Their self-driven nature enhances energy efficiency and device autonomy.^5^ Additionally, these PDs simplify system design by eliminating the need for external power sources. High performance, low cost, and stability make them ideal for use as photodetectors in advanced optoelectronic applications.

Photodetectors that operate in the visible region (400–700 nm) are critical components in a wide range of optoelectronic systems, including optical imaging, environmental monitoring, biological diagnostics, and optical communication. Organic semiconductors have garnered significant attention as potential alternatives to traditional inorganic systems due to their solution processability, mechanical flexibility, tunable band gaps, and low fabrication costs.^5^ Coumarins are a class of heterocyclic compounds known as benzopyrones, characterized by a fused benzene and α-pyrone ring system. Their structural versatility enables the substitution of functional groups at various positions, allowing for a broad spectrum of chemical modifications and diverse applications. Coumarins are excellent electron donors with extended π-conjugation and relatively low oxidation potentials, making them highly suitable for optoelectronic and photonic applications. Naturally occurring in numerous plant species, coumarin derivatives exhibit significant biological activity and are widely utilized in biomedical fluorescence labeling.^6^ Due to their intense fluorescence emission, large Stokes shifts, and high quantum yields, coumarins have gained prominence in the development of light-emitting devices,^7^ dye-sensitized solar cells (DSSCs),^8^ photodetectors, and laser-active media. They have also been employed in reversible optical data storage, brightening agents, and optical switches.^9^ The ability to fine-tune their photoelectric properties through chemical modification has made coumarins and their derivatives highly attractive for organic semiconductor materials, including applications in solar energy harvesting and organic light-emitting diodes (OLEDs).^10^ Notably, Seo *et al.*^11^ demonstrated the photovoltaic efficiency of coumarin dyes incorporating a low bandgap ethylenedioxythiophene (EDOT) chromophore, reporting a fill factor of 0.70 and a power conversion efficiency of 6.07% under AM 1.5 illumination, highlighting their potential as effective electron donors in semiconductor photocells and phototransistors.

Coumarin derivatives are particularly appealing for optoelectronic applications because of their high visual absorption, inherent photoluminescence, and good charge transport properties. Coumarins, a kind of benzopyrone molecule, are well-known for their strong π-conjugation, outstanding photoabsorbance, and electron-donating capabilities, making them great candidates for visible light photodetectors. The coumarin derivative functions as a photoactive medium, absorbing visible light and generating charge carriers that contribute to photocurrent upon illumination.^12^

Despite its promise, extensive studies on the optoelectronic characteristics, manufacturing procedures, and performance of Al/4-FMCSB/ITO visible light detectors have not been conducted. In this article, we describe the fabrication and characterization of a visible photodetector using a Schiff base based on a coumarin derivative (4-FMCSB) as the active light-absorbing layer, which can be utilized in a wide range of optoelectronic systems. Because of its advantageous optical absorption, molecular structure, and charge-transport properties, the coumarin-derived Schiff base (4-FMCSB) is essential as the active layer in the current photodetector. Strong absorption in the visible spectrum, high molar extinction coefficients, and superior photostability are well-known characteristics of coumarin-based compounds, which make them good options for visible-light photodetection.

Additionally, an internal built-in electric field is produced at the interfaces by the asymmetric molecular structure and appropriate energy-level alignment of 4-FMCSB with the Al and ITO electrodes, allowing for self-powered (zero-bias) photodetection. Low dark current and enhanced detectivity are the results of this integrated field's assistance in the separation and extraction of photogenerated carriers without the need for external bias.

## 2 Results and discussion

### 2.1 Synthesis and characterization of coumarin-based Schiff base 4-FMCSB

**Scheme 1:**
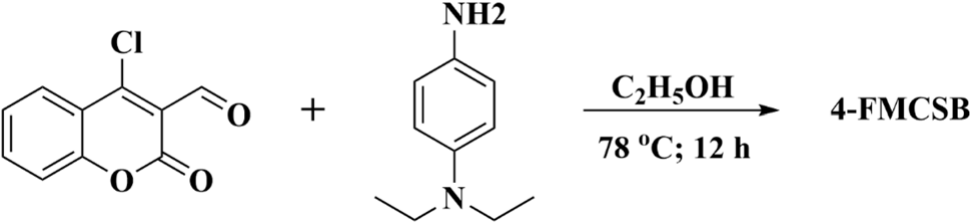
Synthesis of target Schiff base compound 4-FMCSB.

As shown in Scheme 1, the Schiff base 4-FMCSB was successfully synthesized *via* the condensation of compound II with *N*,*N*-diethyl-*p*-phenylenediamine. The complete synthetic route is outlined in Scheme S1. The product was confirmed by ^1^H- and ^13^C-NMR (Nuclear Magnetic Resonance Spectroscopy), FT-IR (Fourier Transform Infrared Spectroscopy), and MALDI-TOF-MS (MALDI-TOF-Mass Spectrometry) analyses, with the corresponding spectra provided in Fig. S1–S6.

The Schiff base 4-FMCSB was synthesized *via* a two-step synthetic route starting from 4-hydroxycoumarin (I), as shown in Scheme 1. In the first step, 4-chloro-2-oxo-2*H*-chromene-3-carbaldehyde (II) was synthesized from I through the Vilsmeier–Haack reaction. Subsequently, II was coupled with *N*,*N*-diethyl-*p*-phenylenediamine in absolute ethanol at room temperature to afford the Schiff base 4-FMCSB with a yield of 92%. The structure of 4-FMCSB was confirmed by spectroscopic characterization using ^1^H NMR, ^13^C NMR, FT-IR, and MALDI-TOF-MS mass spectrometry measurements. The structural analysis of 4-FMCSB using FT-IR measurement is depicted in Fig. S3. The FT-IR spectrum of 4-FMCSB provides clear evidence for the successful formation of the Schiff base. A strong absorption band observed at 1728 cm^−1^ corresponds to the lactone carbonyl (C=O), while the peak at 1079 cm^−1^ is assigned to the C–O–C stretching of the coumarin moiety. The formation of the imine linkage is confirmed by the appearance of a distinct band at 1612 cm^−1^, corresponding to the *ν*_C=N,_ indicating the successful condensation of the amine and aldehyde functional groups. Aliphatic C–H stretching bands are evident at 2971, 2934, and 2893 cm^−1^, indicating the presence of diethylamino substituents. Furthermore, bands at 1239 and 1180 cm^−1^ are attributed to C–N stretching vibrations. The aromatic C=C stretching vibrations are identified at 1572 and 1504 cm^−1^, and the out-of-plane C–H bending of the aromatic ring appears at 828 and 792 cm^−1^.

The ^1^H and ^13^C NMR spectra of 4-FMCSB confirmed the successful formation of Schiff base 4-FMCSB. The ^1^H NMR showed the disappearance of the aldehydic proton signal at *δ* 10.32 ppm, and the emergence of one sharp singlet at *δ* 8.92 ppm corresponding to the formation of azomethine (–CH=N) proton. Additionally, the ^13^C NMR spectra indicated the absence of an aldehydic carbon signal at *δ* 186.5 ppm, further supporting the formation of the imine linkage in 4-FMCSB. Furthermore, the ESI-MS spectrum supports the molecular structure, showing a molecular ion peak at *m*/*z* = 320.1448, which is consistent with the calculated *m*/*z* for 4-FMCSB, *i.e.*, C_20_H_20_N_2_O_2_, of 320.3842 (Fig. S6), thereby confirming the successful synthesis of the target Schiff base derivative 4-FMCSB. The ^1^H and ^13^C NMR spectra of intermediate II and Schiff base 4-FMCSB are presented in Fig. S1–S4.

### 2.2 Thermal stability

**Fig. 1:**
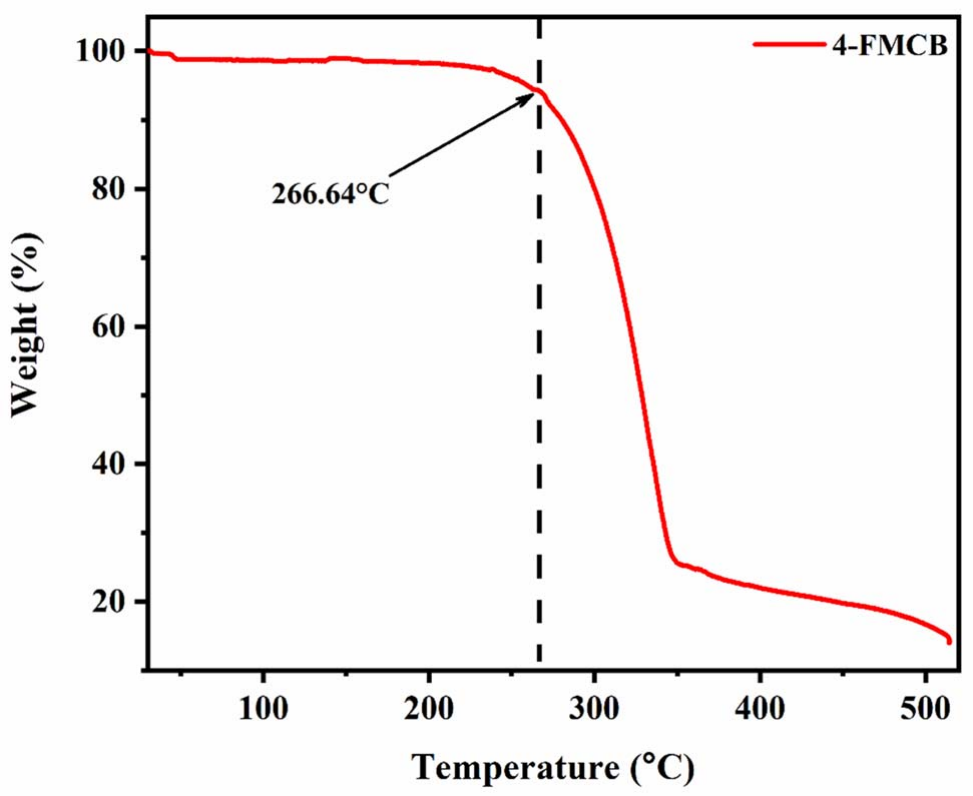
TGA curve of 4-FMCSB in nitrogen atmosphere (heating rate: 10 °C min^−1^).

The thermal stability of 4-FMCSB was assessed using TGA (thermogravimetric analysis), with a temperature range of 30 to 510 °C and a heating rate of 10 °C min^−1^. As shown in Fig. 1, 4-FMCSB exhibits good thermal stability, with a thermal decomposition temperature (*T*_d_) of 266.64 °C at a 6% loss of initial weight. 4-FMCSB shows a sharp weight loss on the TGA curve, indicating it undergoes a significant decomposition stage with good thermal stability. This is consistent with the melting point of 4-FMCSB, which was observed at 266.9 °C.

### 2.3 UV-vis diffuse reflectance spectroscopy (DRS)

**Fig. 2:**
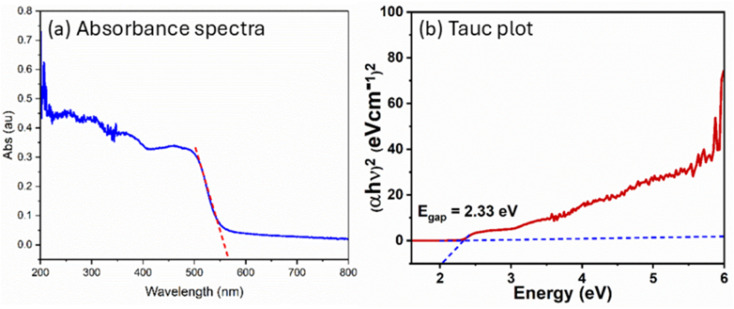
(a) Solid state absorption spectrum of 4-FMCSB; (b) Tauc plot of 4-FMCSB.

The optical and electronic properties of 4-FMCSB were investigated using UV-vis diffuse reflectance spectroscopy (DRS). Fig. 2 presents the DRS spectrum of solid 4-FMCSB. The optical band gap energy (*E*_gap_) value was calculated using the Kubelka–Munk equation in conjunction with the Tauc plot method.^13,14^ The Tauc method is used to calculate the effective energy band gap using the following eqn (1):1
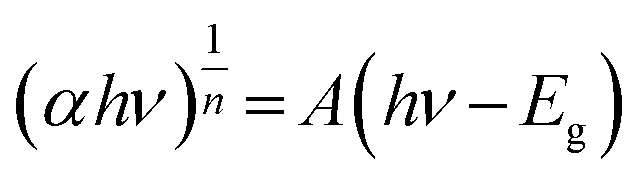
where, *α* = absorption coefficient, *h* = Plank's constant, *ν* = frequency, *E*_g_ = optical band gap, *A* = constant Tauc parameter.

In 4-FMCSB, *n* = 1/2, which refers to a direct transition due to the strong localized nature of π to π* transition. Plotting the dependence of (*αhν*)^2^ on photon energy (*hν*) will give a straight line, and the *y* intercept gives the value of the optical band gap. The slope of the fitting gives *A*.

The constant *A* includes information on the convolution of HOMO and LUMO states and on the matrix element of the optical transition, which quantifies the effectiveness of a photon to excite an electron across the band gap. Moreover, *A* is highly dependent on the character of the bonding. Upon linear fitting of the Tauc plot, the value of *A* is calculated to be 9.262 cm^−2^ eV. It suggests a relatively steep photon absorption onset and a more defined transition from HOMO to LUMO. This correlates with high charge carrier mobility.^15,16^ The calculated band gap energy, *E*_gap_, was found to be 2.33 eV, corresponding approximately to a wavelength of 568 nm, as shown in Fig. 2(a) and (b).

**Fig. 3:**
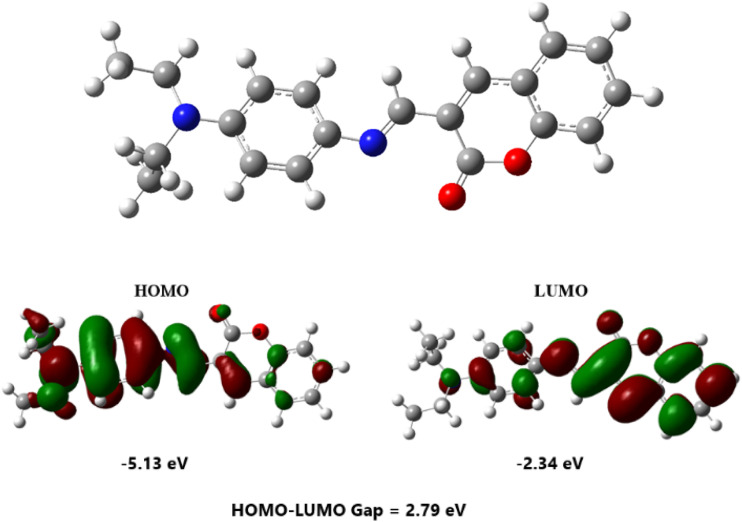
HOMO–LUMO energy gap.

The geometry of the molecule is optimized using the density functional theory (DFT) method with the B3LYP hybrid functional and the 6-311G(d,p) basis set. The calculated HOMO–LUMO energy gap was found to be 2.79 eV, as shown in Fig. 3, which is in good agreement with the experimental energy gap. This result confirms that the synthesized material 4-FMCSB is responsive to visible light, indicating its potential applicability as a visible-light-active photodetector.

### 2.4 FESEM and EDX analysis

**Fig. 4:**
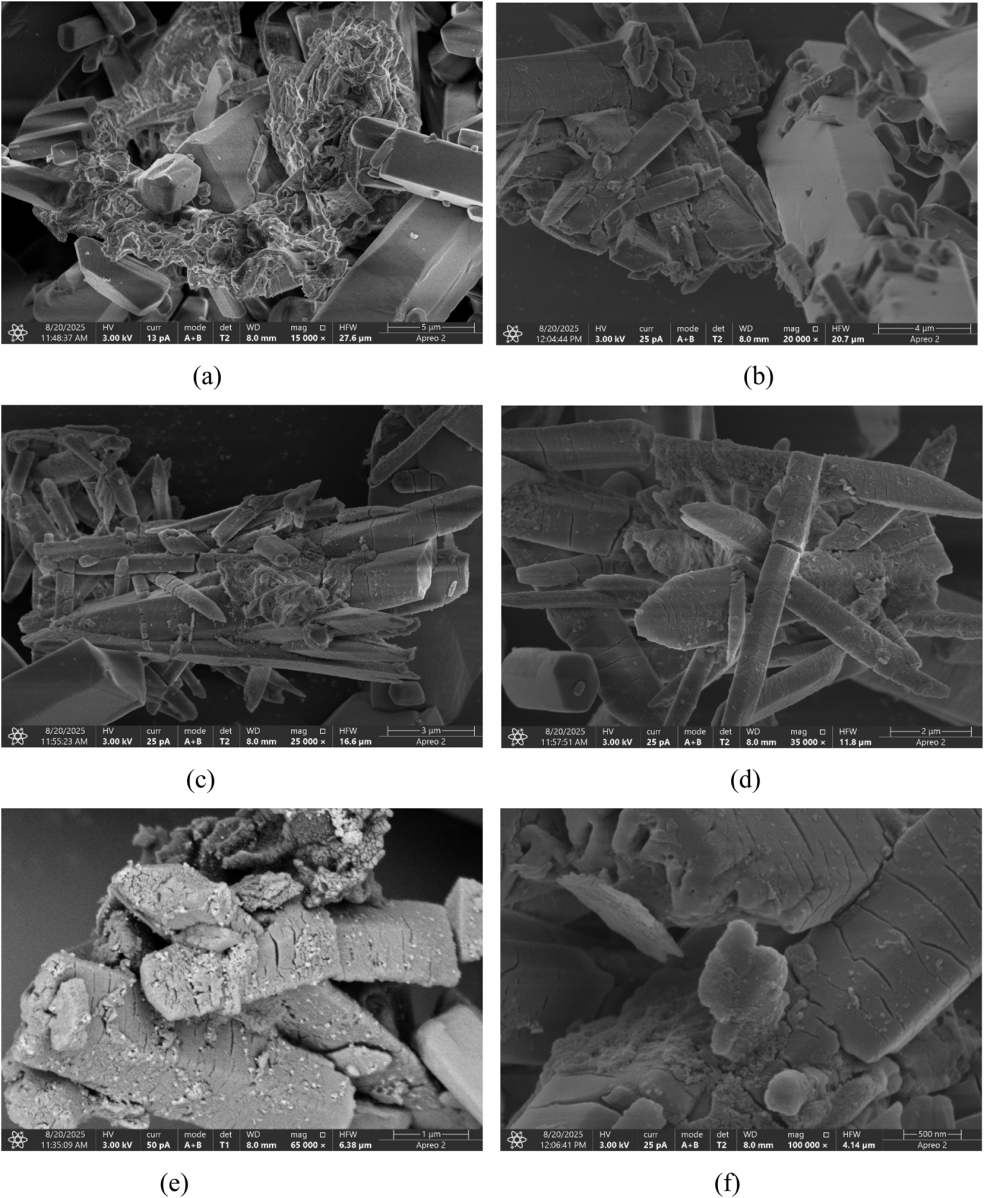
FESEM micrographs of the coumarin derivative at different magnifications: (a) 15k×, (b) 20k×, (c) 25k×, (d) 35k×, (e) 65k×, and (f) 100k×, showing dense, plate-like crystallites and a uniform surface morphology.

FESEM (Field Emission Scanning Electron Microscopy) was employed to analyze the surface morphology and microstructure of the synthesized coumarin derivative 4-FMCSB. Fig. 4(a–f) show micrographs at 15–100k× magnifications, revealing both overall surface uniformity and nanoscale features. At lower magnifications [Fig. 4(a) and (b)], the sample exhibits a compact, uniform morphology consisting of densely packed microcrystals with minimal porosity, indicating consistent nucleation and growth, and a homogeneous film suitable for electronic and optical applications.

At higher magnifications [Fig. 4(c–f)], rod-like and plate-shaped crystallites become visible, reflecting anisotropic molecular packing from π–π stacking and hydrogen bonding (–C=O, –NH–). Finely stacked microcrystalline domains with sharp edges confirm the compound's semi-crystalline nature. The absence of impurities indicates high purity, while the ordered morphology supports efficient charge transport and strong intermolecular coupling, ideal for optoelectronic and sensing applications.

**Fig. 5:**
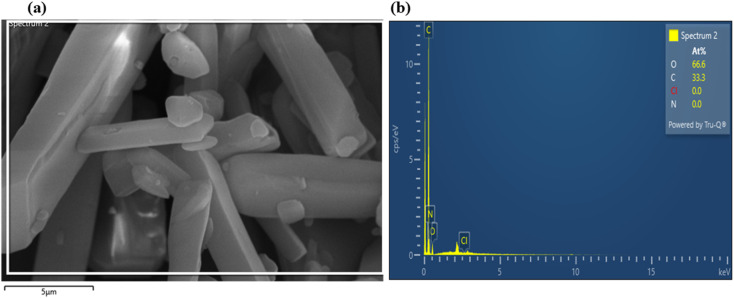
(a) FESEM image of coumarin derivative 4-FMCSB with EDX scan area, and (b) corresponding EDX spectrum.

The EDX (Energy Dispersive X-ray Spectroscopy) spectra of the scanned regions are shown in Fig. 5(a) and (b). Quantitative analysis reveals 66.6 wt% C and 33.3 wt% O, yielding an atomic ratio (C : O ≈ 2 : 1) consistent with that of coumarin-based molecules. Minor carbon variations may arise from molecular substitution or surface oxidation. Oxygen enrichment suggests the presence of additional hydroxyl, carbonyl, or ether groups, possibly resulting from synthesis or mild surface oxidation. Nitrogen (C_20_H_20_N_2_O_2_) was not detected due to low X-ray yield in light elements. No heavy or foreign elements were observed, confirming the high purity of the sample. Overall, EDX results, aligned with FTIR and UV-Vis data, verify the formation of oxygenated aromatic structures characteristic of the coumarin derivative.

**Fig. 6:**
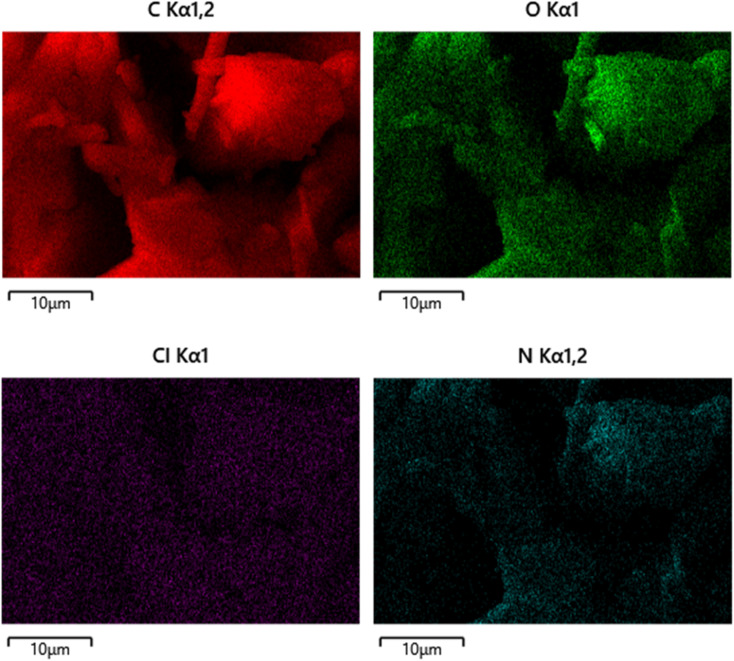
EDX elemental mapping of the coumarin derivative showing uniform distribution of carbon and oxygen throughout the surface, confirming the homogeneity and purity of the synthesized compound 4-FMCSB.

The EDX mapping (Fig. 6) shows homogeneous elemental dispersion (C, O, N) across the surface, verifying that the coumarin derivative is chemically uniform and not phase separated. The minor Cl signal suggests trace impurity. Overall, the EDX confirms that the composition is consistent with the expected formula C_20_H_20_N_2_O_2_, with no significant contamination.

### 2.5 Structural analysis (XRD)

**Fig. 7:**
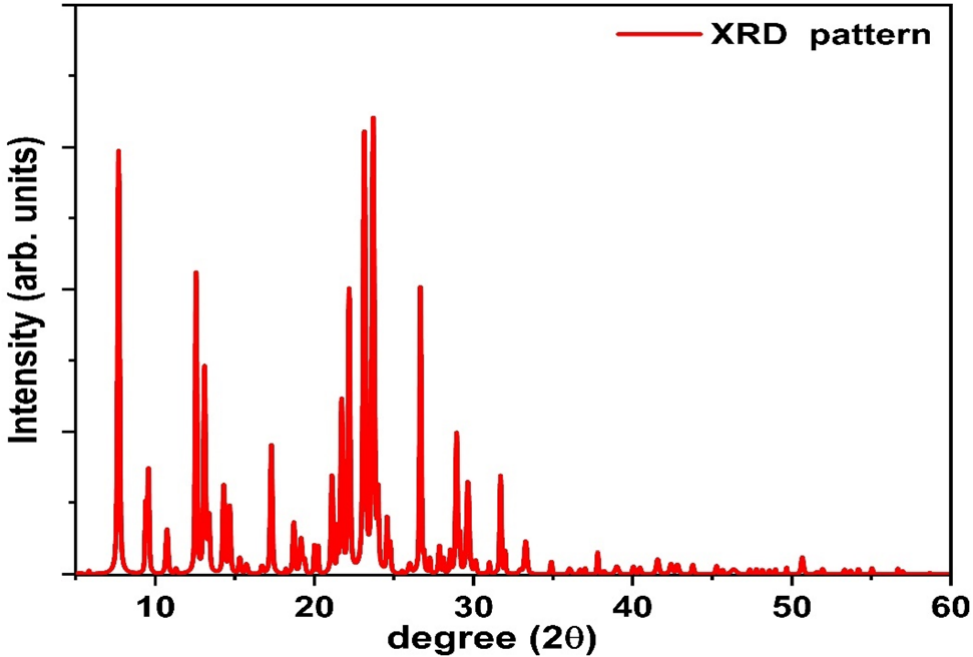
XRD pattern of coumarin derivative 4-FMCSB.

The crystalline nature of the synthesized coumarin derivative 4-FMCSB (C_20_H_20_N_2_O_2_) was investigated using X-ray diffraction (XRD), as shown in Fig. 7. The diffraction pattern was recorded in the 2*θ* range of 5–60°. A series of sharp and well-defined diffraction peaks is observed, indicating that the material exhibits a predominantly crystalline rather than amorphous nature. Such crystallinity is highly desirable for optoelectronic applications, as it generally facilitates improved charge transport by reducing energetic disorder and localized trap states.

Due to the absence of a corresponding JCPDS card for this newly synthesized compound, direct phase identification was not possible. However, the presence of multiple intense diffraction peaks, particularly in the 2*θ* range of 6–35°, suggests a high degree of molecular ordering, which can be attributed to strong π–π stacking and intermolecular interactions among the aromatic rings and heteroatoms in the coumarin-based Schiff base framework. These interactions promote supramolecular organization, which is known to enhance carrier delocalization and mobility in π-conjugated organic materials. Prominent diffraction peaks observed at approximately 7.7°, 12.6°, 23.1°, 23.7°, and 26.9° are indicative of periodic molecular arrangements within the crystal lattice. The relatively strong diffraction intensity at lower 2*θ* angles corresponds to larger interplanar spacings, as described by Bragg's law, and is characteristic of organic materials with extended conjugation and planar molecular stacking. Such large *d*-spacings facilitate efficient exciton diffusion and charge separation, which are critical for photodetection under low-field or zero-bias conditions.

At higher diffraction angles (>35°), the diffraction intensity significantly decreases, which is typical for organic molecular systems composed of light elements. This behavior further confirms the organic nature and phase purity of the synthesized 4-FMCSB material, with no detectable crystalline impurities. Overall, the highly crystalline nature and hierarchical molecular ordering observed in the XRD pattern are expected to play a crucial role in the photodetector performance. Improved molecular packing enhances exciton diffusion, reduces trap-assisted recombination, and promotes efficient charge transport, thereby contributing to the observed high responsivity, detectivity, and stable zero-bias photoresponse of the Al/4-FMCSB/ITO device.

The overall crystalline nature and molecular ordering of the 4-FMCSB thin film are expected to play a significant role in determining the device performance. Improved structural ordering reduces defect-assisted recombination and suppresses dark current, thereby contributing to enhanced photoresponsivity, detectivity, and faster temporal response observed in the fabricated photodetector. Similar correlations between structural ordering and enhanced optoelectronic performance have been reported in recent studies on thin-film photodetectors and semiconductor materials.^17,18^ Thus, the XRD pattern serves not only as a structural fingerprint of the novel coumarin derivative but also provides critical insight into its suitability for self-powered photodetection applications.

## 3 Fabrication of photodetector based on coumarin derivative 4-FMCSB

**Fig. 8:**
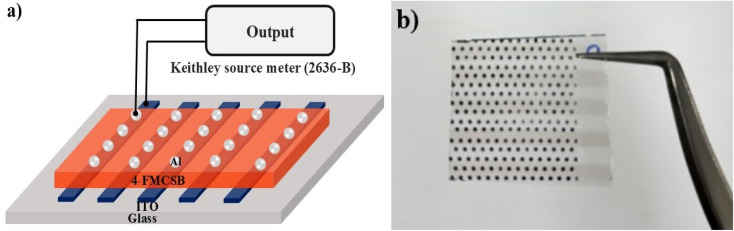
Visible photosensor: (a) schematic representation of the fabricated diode, (b) fabricated diode.

A spin coating process was used to deposit the coumarin-based Schiff base (4-FMCSB) thin films onto patterned ITO-coated glass substrates. The substrates were cleaned using a standard protocol involving 10-minute ultrasonication in acetone and isopropyl alcohol (IPA), followed by nitrogen gas drying and a 15-minute UV-ozone treatment. After cleaning, the 4-FMCSB solution was deposited onto the ITO-coated glass substrate using a HOLMARC spin coater (Model: HO-TH-5) at 3000 rpm for 30 s. The film was then preheated at 250 °C for 5 minutes to evaporate solvents and remove organic contaminants. This process was repeated for multiple layers until the desired thickness was achieved. To fabricate the photosensor, 100 nm thick aluminum contacts were grown onto the film using a 760 µm diameter dotted shadow mask. Thermal evaporation was used to deposit the material at a steady rate of 5 Å s^−1^, monitored by a quartz crystal oscillator. The schematic representation of the fabricated Al/4-FMCSB/ITO visible photo detector is shown in Fig. 8.

To evaluate the device's electrical properties, current–voltage (*I*–*V*) measurements were taken with a Keithley 2636-B source meter. The performance under illumination was assessed with optimum light at a wavelength of 540 nm and an intensity of 0.28 mW cm^−2^. For transient photoresponse investigations, optical signals were modified, and the current responses were measured using the same source meter.

**Fig. 9:**
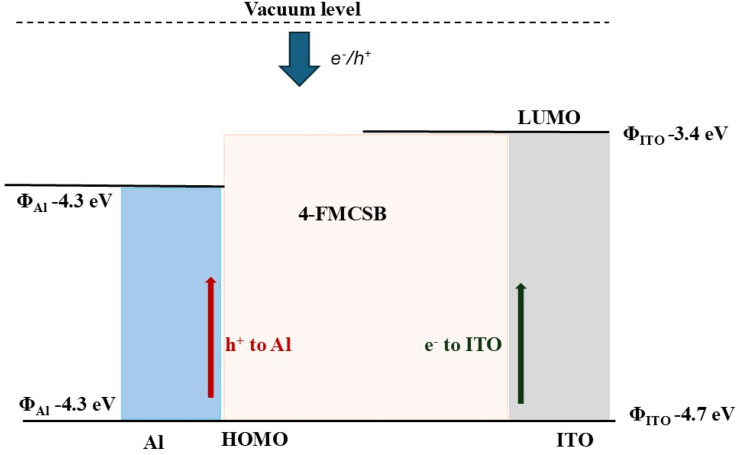
Energy level diagram of the Al/4-FMCSB/ITO photodetector.

Fig. 9 illustrates a qualitative energy band diagram of the Al/4-FMCSB/ITO photodetector under equilibrium (zero bias). Due to the difference in work functions of Al (≈4.3 eV) and ITO (≈4.7 eV), an internal built-in electric field is established across the 4-FMCSB active layer. Upon illumination, photogenerated electrons and holes are efficiently separated and transported toward Al and ITO electrodes, respectively, enabling self-powered photodetection without external bias. Although Al and ITO possess different work functions, upon forming the Al/4-FMCSB/ITO heterostructure and reaching equilibrium, a common Fermi level is established across the device. The work function difference induces band bending within the organic layer, resulting in an internal built-in electric field that enables efficient separation of photogenerated charge carriers under zero external bias. The energy level diagram is schematic and intended to qualitatively illustrate the operating mechanism, as direct experimental determination of HOMO–LUMO levels for the novel 4-FMCSB material is currently unavailable.

### 3.1 Current–voltage characteristics

**Fig. 10:**
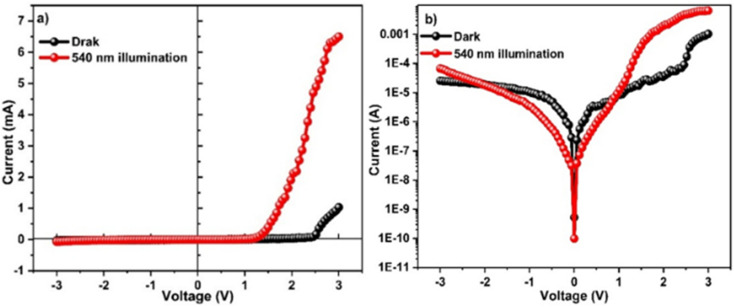
Current–voltage characteristics under dark and illumination at (a) linear scale and (b) log scale.

The *I*–*V* behavior of the Schiff base of coumarin (4-FMCSB)-based photodetector was studied under dark conditions and 540 nm light illumination across a voltage range from −3 to +3 V. Fig. 10(a) and (b) depict the *I*–*V* characteristics of the Al/4-FMCSB/ITO configuration. The observed rectification ratios were 4.05 × 10^1^ and 2.55 × 10^2^ under dark and illumination conditions, respectively. Rectification is produced by the creation of intrinsic defects within the active layer thin films and at the metal contact interface. The narrowing of energy bands at low forward bias causes a significant rise in voltage current density, resulting in the dominance of a space-charge-limited current (SCLC) mechanism.^19,20^

Under dark conditions, the device exhibits a resistance of 1.18 × 10^5^ Ω. The resistance decreases to 4.45 × 10^4^ Ω with illumination, indicating enhanced carrier generation due to light absorption. Fabricated detector shows a clear photoresponse, where light exposure generates charge carriers that increase conductivity and lower resistance. This confirms the device's effective sensitivity to visible light, as expected for a photodetector. The ideality factor and other junction characteristics were calculated using the thermionic emission model. A diode's current (*I*) is expressed by^21^2
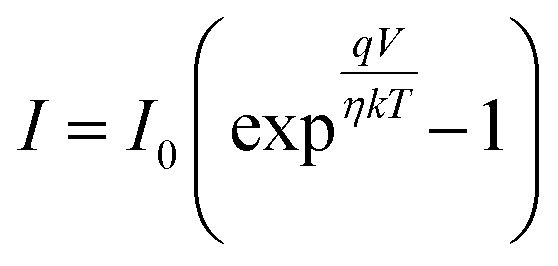
where *T* is the temperature in Kelvin, *η* is the ideality factor, *k* is the Boltzmann constant, *q* is the elementary charge, and *I*_0_ is the saturation current. Eqn (2) may be represented as follows:3
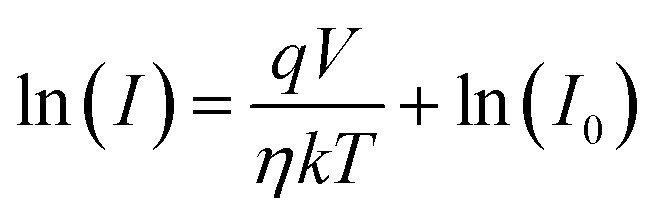


The observed Ideality factor (*η*) for the device under dark conditions is 1.53, and under illumination is 1.61. Under illumination, the ideality factor is increased compared to dark conditions. The increase in *η* under illumination in our device reflects the influence of photo-induced recombination and defect activity, which is common in organic and hybrid photodetectors. Under illumination, the generation of additional charge carriers can lead to increased recombination *via* defect states, such as trap-assisted (Shockley–Read–Hall) recombination, especially at interfaces or within the bulk of the active layer. This non-ideal recombination contributes to a higher ideality factor, deviating from the ideal diode behavior. This phenomenon is well-documented in the literature. For instance, studies on perovskite solar cells have shown that illumination can activate defect states, leading to increased recombination and, consequently, a higher ideality factor.^22^ In particular, research has indicated that under light exposure, the ideality factor can increase due to enhanced monomolecular recombination processes associated with defect states.

### 3.2 Photo response analysis

**Fig. 11:**
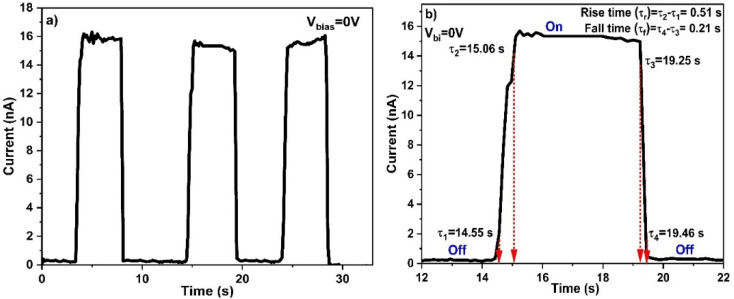
Transient photocurrent measurement of fabricated photosensor at 0 bias (self-biased), (a) speed of response (full cycle), and (b) rise and fall time (single cycle).

The photoresponse of the Al/4-FMCSB/ITO device was investigated under monochromatic visible-light illumination (*λ* = 540 nm) at an optimized power density of 0.28 mW cm^−2^. Fig. 11 illustrates the transient photocurrent characteristics of the photodetector operated under zero external bias (self-powered mode). The current–time profile exhibits a rapid and repeatable increase in photocurrent upon illumination with light, followed by a swift decay when the light source is turned off. The stable and reproducible on–off switching behavior over multiple cycles confirm the excellent sensitivity, reliability, and operational stability of the device toward visible-light irradiation.

The rapid photocurrent generation upon illumination can be attributed to efficient photon absorption in the coumarin-derived Schiff base thin film, followed by exciton generation and effective charge separation at the Al/4-FMCSB and 4-FMCSB/ITO interfaces. The built-in electric field arising from the asymmetric metal–semiconductor contacts enables carrier separation and transport even in the absence of an external bias, validating the self-powered nature of the device.

To quantitatively evaluate the photodetector performance, key photosensing parameters such as photoresponsivity (*R*), specific detectivity (*D*), linear dynamic range (LDR), and transient response speed were calculated. The responsivity, which measures the electrical output per unit incident optical power, was calculated using eqn (4). The sensors' sensitivity to light exposure is quantified as responsivity, *R* (A W^−1^), computed as follows:^23^4
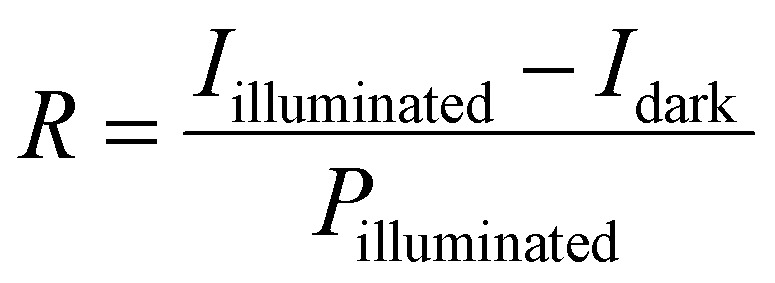
where *I*_illuminated_, *I*_dark_, and *P*_illuminated_ denote the photocurrent under illumination, the dark current, and the light intensity incident on the active portion of the device.

The fabricated device exhibits a responsivity of 12.6 mA W^−1^ at zero bias, which is notable for an organic photodetector operating in a self-powered mode. Although higher responsivity values have been reported in inorganic chalcogenide thin films subjected to time-dependent laser irradiation, such devices typically require external biasing and post-deposition modification. In contrast, the present device achieves appreciable responsivity without any external power source, making it highly attractive for low-power optoelectronic applications.^24,25^ The specific detectivity (*D*), a crucial figure of merit for weak-light detection, was estimated using eqn (5), assuming shot-noise-limited performance.^23^5
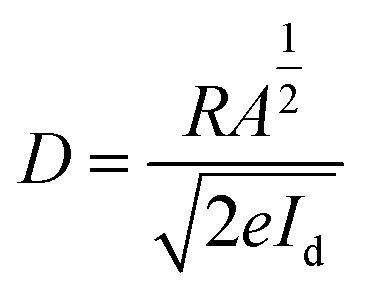
*R* represents responsivity, *I*_d_ is the dark current, *A* is the effective area that contributes to light absorption in the device, and e is an electron's charge. A detectivity of 9.31 × 10^10^ jones was obtained at zero bias, which is primarily attributed to the extremely low dark current of the device. The suppression of dark current under self-bias conditions significantly enhances the signal-to-noise ratio, leading to improved detectivity. Furthermore, the linear dynamic range (LDR), which reflects the device's ability to respond linearly over a wide range of illumination intensities, was calculated using eqn (6).^26^6
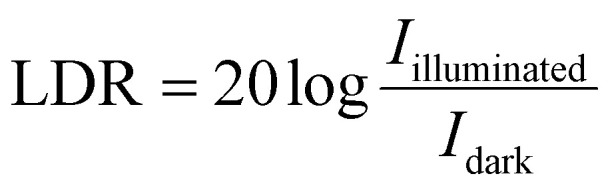


The device demonstrates an LDR of approximately 35.94 dB, indicating a substantial difference between the photocurrent and dark current levels. This wide LDR is advantageous for practical photodetection applications where illumination intensity may vary significantly.

The temporal photoresponse of the device was also analyzed to assess its switching speed, specifically the rise time (*τ*_rise_) and decay time (*τ*_decay_), provide critical insight into the carrier generation, separation, transport, and recombination dynamics within the active layer of the photodetector. The rise time, defined as the time required for the photocurrent to increase from 10% to 90% of its maximum value, was found to be 510 ms, while the fall time (90% to 10%) was 210 ms, as shown in Fig. 11(b). The faster decay compared to the rise time suggests efficient carrier recombination and minimal charge trapping in the active layer. The relatively fast response under zero-bias operation indicates that carrier transport is predominantly governed by the built-in electric field and low trap-assisted recombination, further confirming the suitability of the 4-FMCSB thin film for self-powered visible-light photodetection.

The comparatively shorter decay time suggests that once the incident light is removed, the majority of photogenerated carriers rapidly recombine or are extracted, pointing to low trap-state density and efficient carrier extraction pathways. In contrast, longer rise times often reflect the time required to fill available trap states and establish a steady-state photocurrent under illumination. These dynamics are consistent with reports in other high-performance photodetectors, where fast rise and decay responses have been correlated with efficient charge separation and minimized defect-mediated recombination pathways.^27,28^ Importantly, although many high-speed inorganic photodetectors operate under externally applied bias to enhance internal fields and carrier sweep-out, the current coumarin-derived Schiff-base device achieves competitive temporal response under zero applied bias. This feature underscores the favorable intrinsic properties of the organic active layer and its interfaces with the electrodes, resulting in sufficient built-in fields that facilitate rapid carrier motion and resetting of the conduction state upon light removal. Moreover, the relatively fast photoresponse observed here implies that the device can be potentially useful for applications requiring rapid detection of pulsed or modulated visible-light signals.

The operational stability of the Al/4-FMCSB/ITO photodetector was evaluated through repeated ON–OFF illumination cycles under 540 nm light at zero bias. As shown in Fig. 11, the device exhibits highly reproducible photocurrent responses over multiple switching cycles with negligible variation in peak photocurrent and no observable baseline drift. The stable and repeatable photoresponse confirms the robust nature of the metal–organic interfaces and the reliability of the 4-FMCSB active layer under cyclic illumination conditions. The shelf-life stability of the Al/4-FMCSB/ITO photodetector was not systematically investigated through extended storage aging tests in the present study. However, the device exhibits stable and reproducible photoresponse under repeated cyclic illumination, indicating robust metal–organic interfaces and intrinsic material stability. Comprehensive shelf-life and long-term environmental stability studies will be the focus of future investigations to further evaluate the practical applicability of the device.

## 4 Conclusion

In conclusion, a visible-light photodetector based on the coumarin-derived Schiff base (4-FMCSB) was successfully synthesized and characterized. A self-powered visible photodetector employing thin films of 4-FMCSB was fabricated, demonstrating a strong rectification ratio of two orders of magnitude. The device operated reliably without any external voltage, confirming its zero-bias functionality. It exhibited rapid photoresponse characteristics, with a rise time of 510 ms and a fall time of 210 ms. Under zero bias, the photodetector achieved a responsivity of 12.6 mA W^−1^, a detectivity of approximately 9.31 × 10^10^ jones, and a linear dynamic range (LDR) of 35.94 dB.

## Conflicts of interest

There are no conflicts to declare.

## Supplementary Material

RA-016-D5RA09436D-s001

## Data Availability

The data supporting this article have been included as part of the supplementary information (SI). Supplementary information: detailed synthetic route and proposed chemical structure of 4-FMCSB is shown in Scheme S1; details of characterization of 4-FMCSB: FT-IR spectra, ^1^H NMR, ^13^C NMR and, MALDI-TOF MS spectrum is shown in Fig. S1–S6. See DOI: https://doi.org/10.1039/d5ra09436d.

## References

[cit1] Singh M., Ambedkar A. K., Tyagi S., Kumar A., Kumar A., Gautam Y. K., Sharma K., Singh B. P. (2023). ACS Omega.

[cit2] Suchikova Y., Nazarovets S., Popov A. I. (2024). Opt. Mater..

[cit3] Cardona M. and Yu P. Y., in Comprehensive Semiconductor Science and Technology, ed. P. Bhattacharya, R. Fornari and H. Kamimura, Elsevier, Amsterdam, 2011, pp. 125–195

[cit4] Silva J. P. B., Vieira E. M. F., Gwozdz K., Kaim A., Goncalves L. M., MacManus-Driscoll J. L., Hoye R. L. Z., Pereira M. (2021). Nano Energy.

[cit5] Altınışık S., Ozdemir M., Kortun A., Zorlu Y., Yalçın B., Koksoy B., Koyuncu S. (2023). ACS Omega.

[cit6] Das S., Indurthi H. K., Saha P., Sharma D. K. (2024). Dyes Pigm..

[cit7] Wu X., Su R., Zhao Y., Ma H., Liu X., Qian L., Han M., Su W., Yu T. (2022). New J. Chem..

[cit8] Evangelista E., de Jesus I. S., Pauli F. P., de Souza A. S., Borges A. d. A., Gomes M. V. S. F., Ferreira V. F., da Silva F. d. C., Melo M. A., Forezi L. d. S. M. (2025). ACS Omega.

[cit9] Cazin I., Rossegger E., Guedes de la Cruz G., Griesser T., Schlögl S. (2020). Polymers.

[cit10] Yakuphanoglu F., Mensah-Darkwa K., Al-Ghamdi A. A., Gupta R. K., Farooq W. A. (2016). Microelectron. Eng..

[cit11] Seo K. D., Song H. M., Lee M. J., Pastore M., Anselmi C., De Angelis F., Nazeeruddin M. K., Gräetzel M., Kim H. K. (2011). Dyes Pigm..

[cit12] Aslan F., Esen H., Yakuphanoglu F. (2019). J. Alloys Compd..

[cit13] Wood D. L., Tauc J. S. (1972). Phys. Rev. B.

[cit14] Ait Baha A., Khossossi N., Lakbita O., Brahmi Y., El Mernissi Y., Aziz T., Benlhachemi A., Bakiz B., Abou Oualid H. (2024). Chem. Phys. Lett..

[cit15] Naik R., Kumar C., Ganesan R., Sangunni K. S. (2011). Mater. Chem. Phys..

[cit16] Naik R., Jena S., Ganesan R., Sahoo N. K. (2014). Phys. Status Solidi B.

[cit17] Priyadarshini P., Senapati S., Bisoyi S., Samal S., Naik R. (2023). J. Alloys Compd..

[cit18] Das S., Senapati S., Pradhan G. K., Varadharajanperumal S., Naik R. (2023). ACS Appl. Nano Mater..

[cit19] Tripathi D. C., Mohapatra Y. N. (2014). J. Appl. Phys..

[cit20] Lettieri S., Pavone M., Fioravanti A., Santamaria Amato L., Maddalena P. (2021). Materials.

[cit21] Agrohiya S., Kumar V., Rawal I., Dahiya S., Goyal P. K., Kumar V., Punia R. (2022). Silicon.

[cit22] Calado P., Burkitt D., Yao J., Troughton J., Watson T. M., Carnie M. J., Telford A. M., O'Regan B. C., Nelson J., Barnes P. R. F. (2019). Phys. Rev. Appl..

[cit23] Salunkhe P., Bhat P., Kekuda D. (2022). Sens. Actuators, A.

[cit24] Giri S., Kumar P. C., Supriya S., Alagarasan D., Naik R. (2025). RSC Adv..

[cit25] Mahapatra L., Kumar P. C., Pradhan P., Alagarasan D., Sripan C., Naik R. (2025). Mater. Adv..

[cit26] Bhat P., Salunkhe P., Kekuda D. (2023). Appl. Phys. A.

[cit27] Das S., Dandsena B., Das K., Supriya S., Kumar P. C., Naik R. (2025). ACS Appl. Nano Mater..

[cit28] Kumar P. C., Kumar J., Sripan C., Naik R. (2025). ACS Appl. Electron. Mater..

