# Supplementary material for: Design and fabrication of a zero-bias visible light photodetector based on a coumarin-derived Schiff base thin film
Source: RSC Adv. 2026 Jan 26;16(6):5455–65. doi: 10.1039/d5ra09436d (PMC12834207; doi:10.1039/d5ra09436d)
Supplement: RA-016-D5RA09436D-s001 [file RA-016-D5RA09436D-s001.pdf]

This journal is © The Royal Society of Chemistry 2026

Design and Fabrication of a Zero-Bias Visible Light Photodetector based on Coumarin-Derived Schiff base thin film.

Ishanki Sharma[a], Jagadish K A[a], Suranjan Shil[b], Dhananjaya Kekuda[a], NV Anil Kumar*[a]

[a] School of Basic Science, Humanities and Management, Manipal Institute of Technology, Manipal Academy of Higher Education, Manipal, India.

[b] Manipal Centre for Natural Sciences, Manipal Academy of Higher Education, Manipal, India.

*nv.anil@manipal.edu

Experimental

Materials and Methods

4-Hydroxycoumarin and N, N-Diethyl-p-phenylenediamine were purchased from Sigma-Aldrich and used without further purification. All the solvents and reagents used were of analytical grade and spectroscopic grade. The progress of the reaction was monitored using alumina sheets precoated with silica gel (Merck, Kieselgel 60, F254), which were used for thin-layer chromatography (TLC).

Measurement and Characterization

Structural Characterization

^{1}H NMR (400 MHz) and 13C NMR (101 MHz) spectra in CDCl3 were recorded at room temperature using a 400 MHz Bruker Avance Neo spectrometer. Tetramethylsilane (TMS) was used as an internal standard.

Fourier-Transform Infrared (FT-IR) Spectroscopy

FT-IR spectra were recorded on a Shimadzu FT-IR spectrometer using the ATR technique in the range of 400 to 4000 cm^{-1}.

UV–vis Measurements

UV–vis-NIR and solid-state reflectance spectra were recorded using the Cary 5000 spectrophotometer, 200–800 nm by Agilent Technology for UV–vis spectra measurements.

Powder X-ray Diffraction (PXRD)

Powder X-ray diffraction (PXRD) measurements were done on a Rigaku ultima IV X-ray diffractometer with Cu Kα X-ray radiation, $\lambda = 1.5406$ Å, $5° \leq 2\theta \leq 60°$.

Field-Emission Scanning Electron Microscope (SEM)

The FE-SEM images were recorded using a Thermo Fisher Apreo 2S with 15-100 kX magnifications.

Thermogravimetric Analysis (TGA)

Thermogravimetric analysis (TGA) was performed on a Shimadzu DTG-60 instrument under a nitrogen atmosphere with a heating rate of 10 °C min^{-1} over a temperature range of 25 to 600 °C using an aluminum pan.

Computational details:

The geometry of the molecule is optimized using the density functional theory (DFT) method with the B3LYP hybrid functional and the 6-311G(d,p) basis set. The calculations were performed using the Gaussian16 software. [1]

Synthesis and Characterization

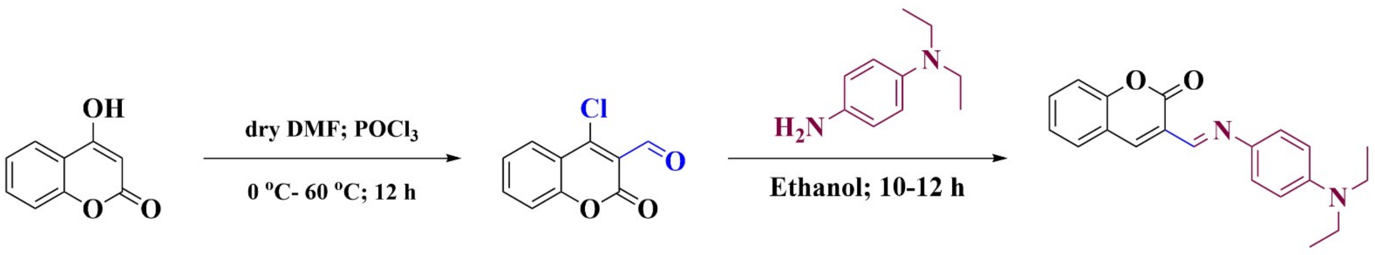

(I)   (II)   4-FMCSB

Scheme S1 Synthetic Route and Proposed Chemical Structure of **4-FMCSB**

Synthesis of 4-Chloro-2-oxo-2*H*-chromene-3-carbaldehyde (II) ($C_{10}H_5ClO_3$)

The starting material, 4-chloro-3-formylcoumarin (II), was synthesized from 4-hydroxycoumarin (I) via the Vilsmeier–Haack reaction, as previously reported in the literature.[2,3] To a cooled solution of dry DMF (5 mL) at 0 °C, Phosphorus oxychloride ($POCl_3$, 5 mL) was added in a single portion. The reaction mixture was subsequently heated to 50 °C and stirred for 30 minutes. Subsequently, a solution of 4-hydroxycoumarin (1.10 g, 6.71 mmol) in dry DMF (10 mL) was added, and the reaction was continued at 60 °C for 12 hours. Upon completion, the mixture was allowed to cool to room temperature and then poured into ice water. The resulting precipitate was collected by

filtration and extracted twice with Dichloromethane. The combined organic layers were dried over anhydrous magnesium sulfate ($MgSO_4$), filtered, and evaporated to afford a pale yellow solid. Yield: 90%. ^1H NMR (400 MHz, $CDCl_3$) δ 10.32 (s, 1H), 8.07 (d, *J* = 8.1 Hz, 1H), 7.67 (t, *J* = 8.4 Hz, 1H), 7.37 (dd, *J* = 23.5, 8.3 Hz, 2H). ^{13}C NMR (101 MHz, $CDCl_3$) δ 186.85, 158.47, 153.55, 153.29, 135.72, 127.68, 125.59, 118.41, 118.22, 117.23. ^{13}C NMR (101 MHz, $CDCl_3$) δ 186.85, 158.47, 153.55, 153.29, 135.72, 127.68, 125.59, 118.41, 118.22, 117.23, 77.36, 77.04, 76.72.

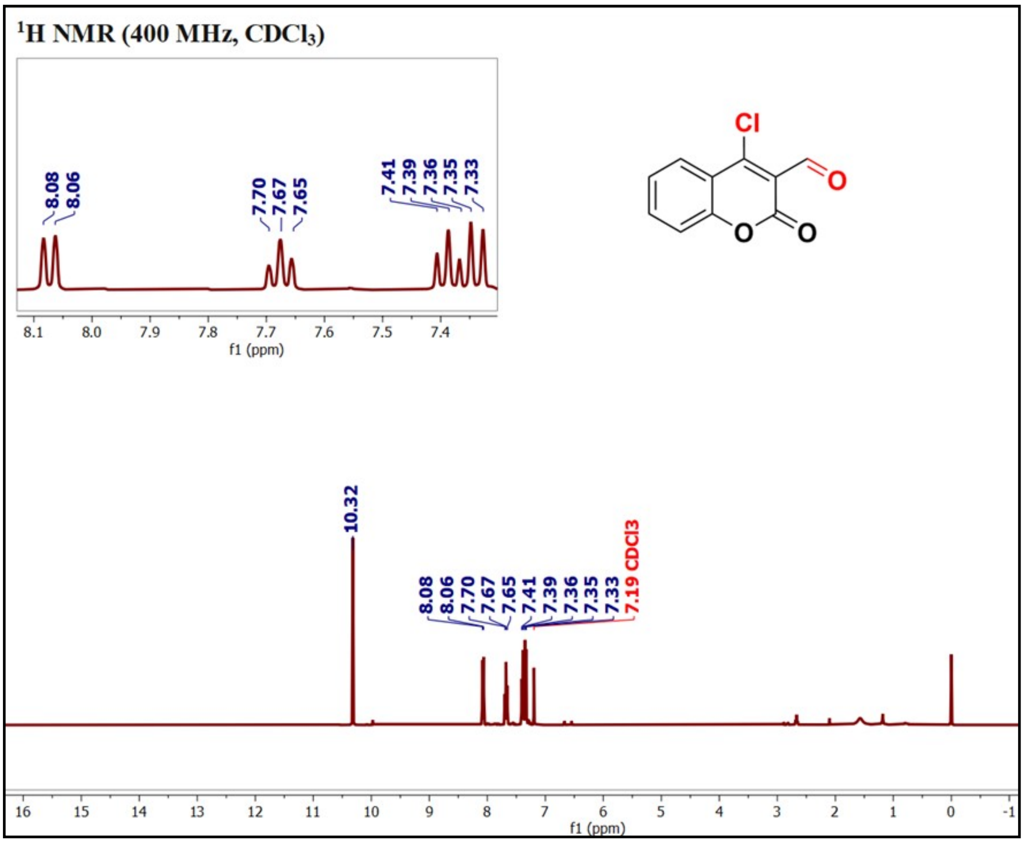

Figure S1 ^1H N.M.R of II

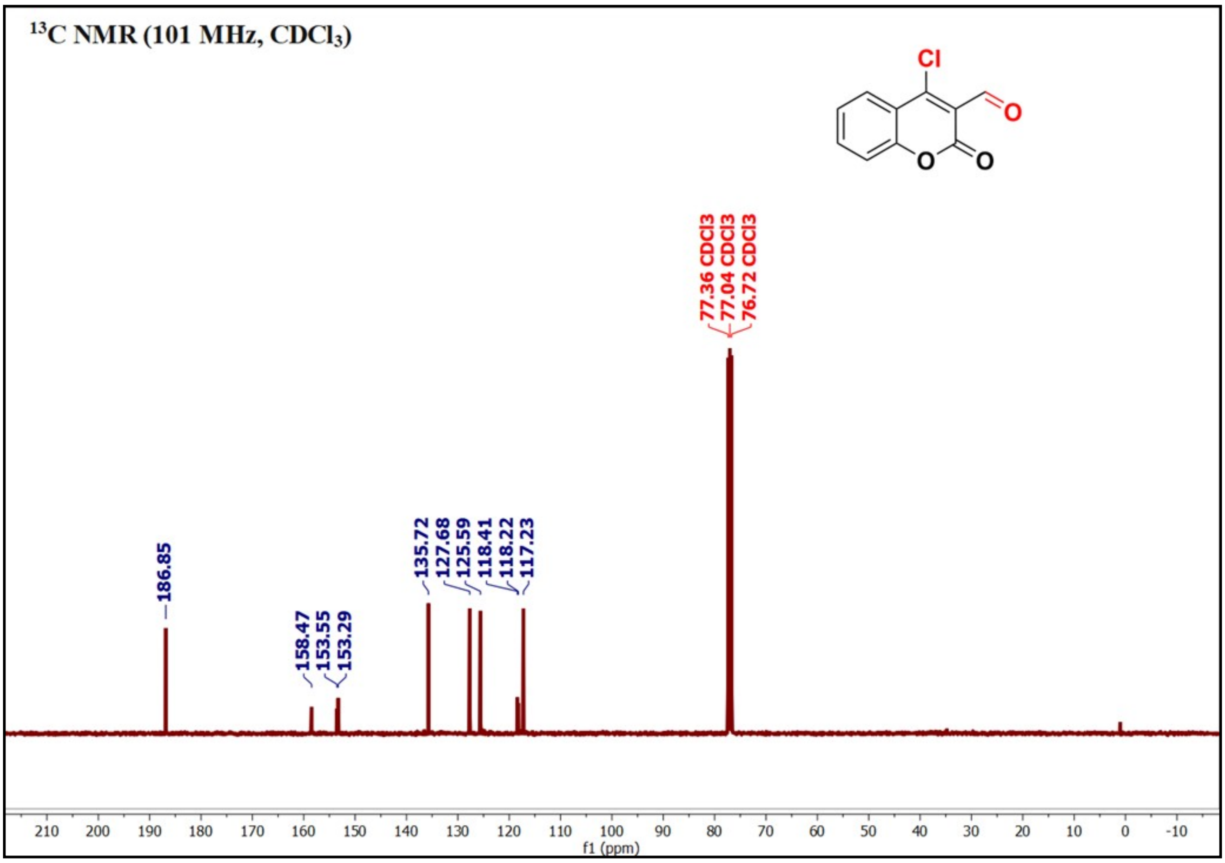

Figure S2 ^{13}C N.M.R of II

Synthesis of (E)-3-(((4-(diethylamino) phenyl) imino) methyl)-2H-chromen-2-one (4-FMCSB)

4-Chloro-3-formylcoumarin (II) (1g, 4.79 mmol) was dissolved in absolute ethanol (15 mL). To this solution, N, N-Diethyl-p-phenylenediamine (0.787 g, 4.79 mmol) was added, and the reaction mixture was stirred at room temperature for 10-12 hours. An orange solid gradually precipitated from the solution. The reaction mixture was then filtered off, and washed with cold ethanol, and dried under ambient conditions. Yield: 92%. FTIR (cm^{-1}): ν (C=O) = 1728 cm^{-1}; ν (C-O-C) = 1079 cm^{-1}; ν (C=N) = 1612 cm^{-1}; ν $(C–H)_{aliphatic}$= 2971, 2934, and 2893 cm^{-1}; ν (C–N) = 1239 and 1180 cm^{-1}; ν $(C=C)_{aromatic}$ = 1572 and 1504 cm^{-1}; and ν $(C\text{-}H)_{bending\ aromatic}$ = 828 and 792 cm^{-1}. ^{1}H NMR (400 MHz, $CDCl_3$) δ 8.92 (s, 1H), 8.70 (d, J = 9.9 Hz, 1H), 8.06 (d, J = 9.5 Hz, 1H), 7.51 (t, J = 9.8 Hz, 1H), 7.43 – 7.31 (m, 2H), 3.52 (q, J = 7.1 Hz, 4H), 1.28 (t, J = 7.1 Hz, 6H). ^{13}C NMR (101 MHz, $CDCl_3$) δ 162.51, 152.40, 146.82, 145.52, 137.81, 131.32, 130.78, 130.07, 125.18, 124.89, 123.59, 120.78, 117.65, 116.38, 104.40, 45.16, 13.08. MALDI-TOF-MS m/z calc. for $C_{20}H_{20}N_2O_2$: 320.3842; found m/z = 320.208 $[M+H]^+$.

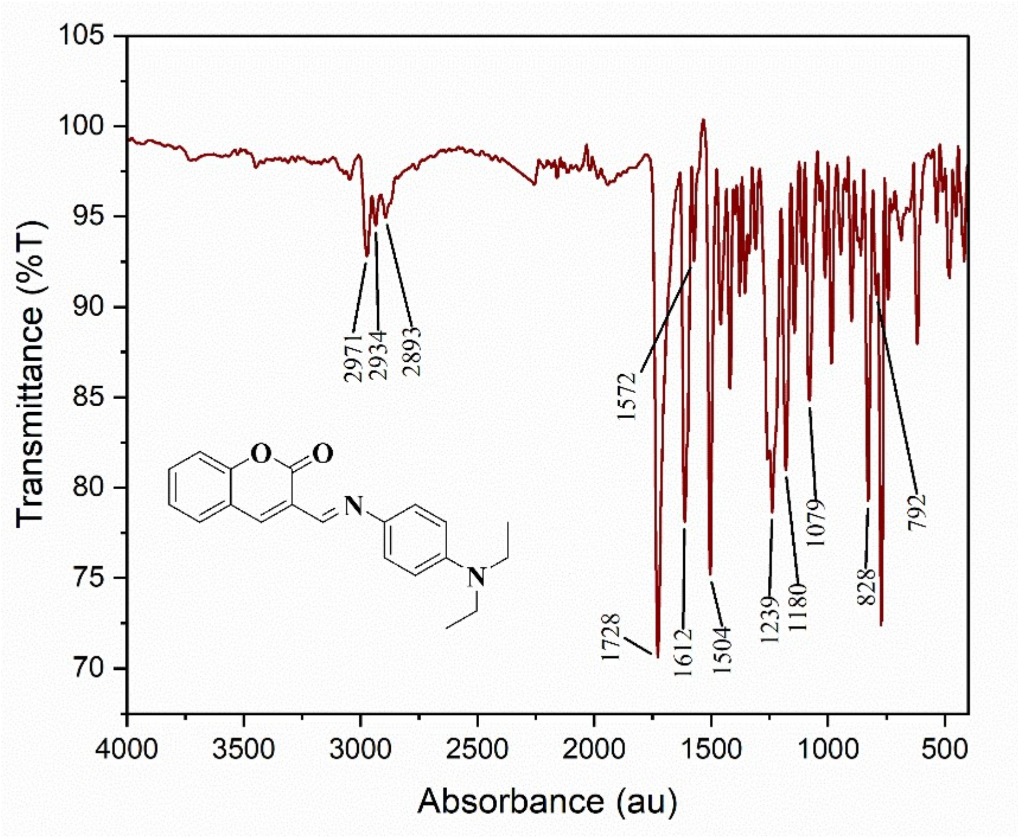

Figure S3 FT-IR Spectra of 4-FMCSB

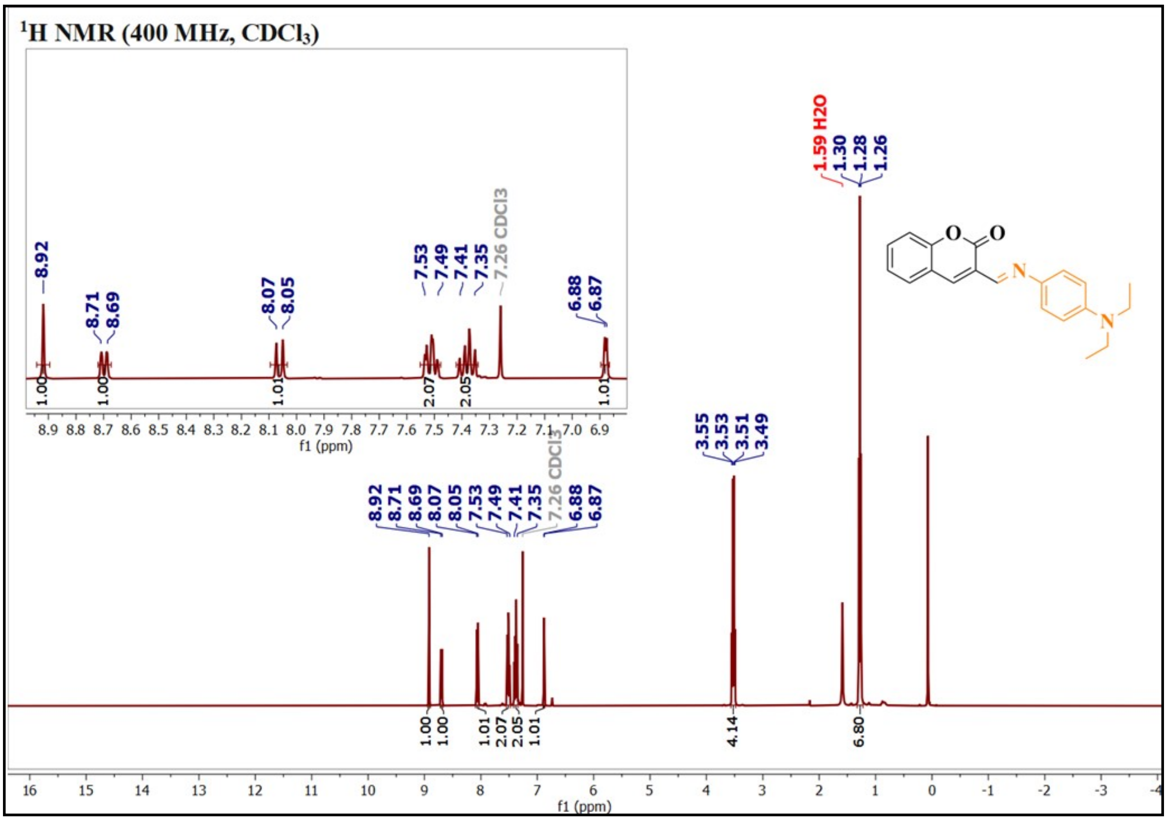

Figure S4 ^{1}H N.M.R of 4-FMCSB

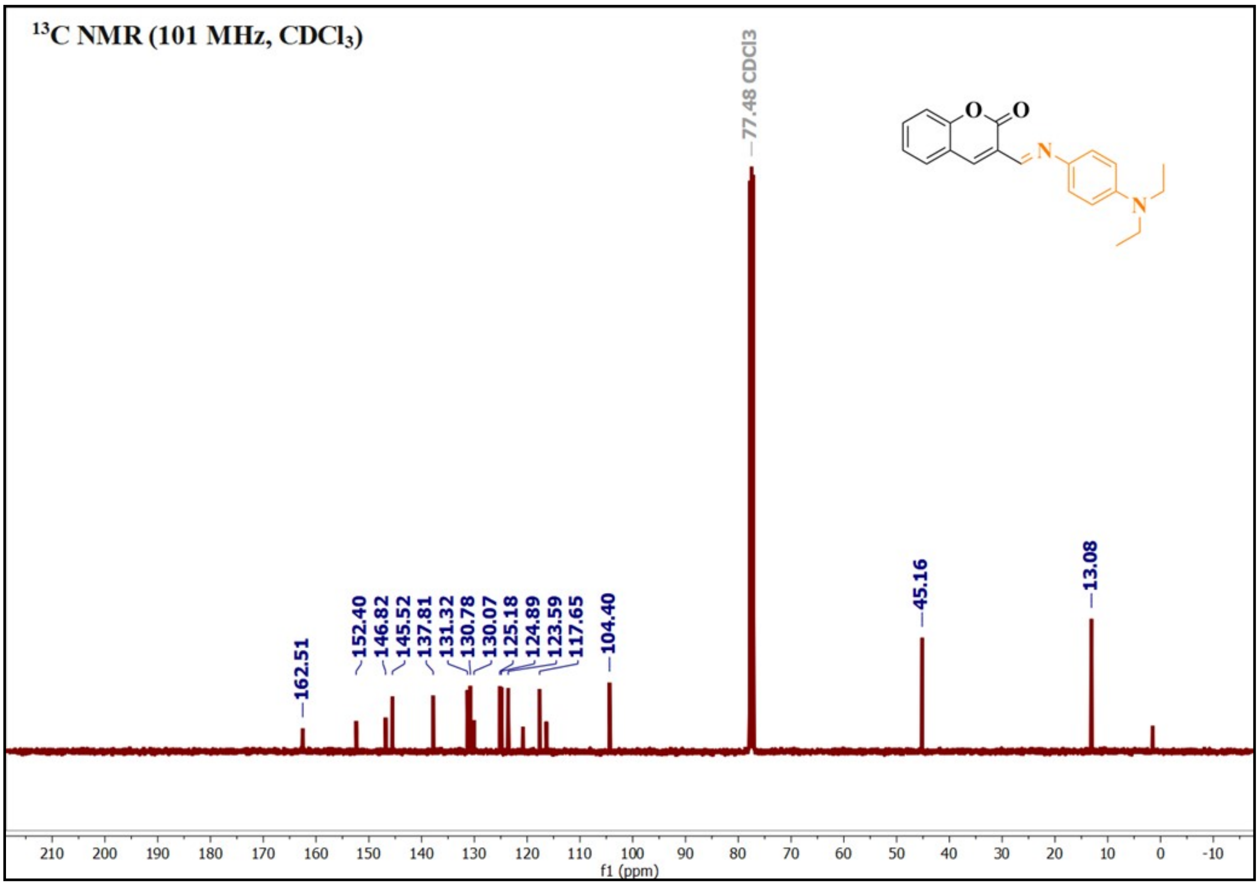

Figure S5 ^{13}C N.M.R of 4-FMCSB

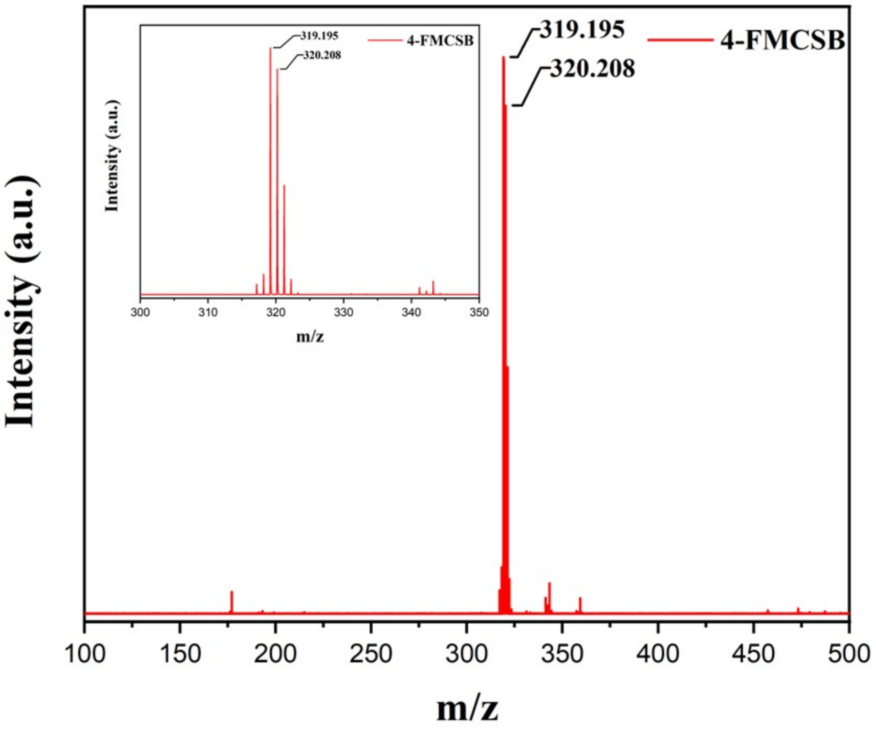

MALDI-TOF-MS, *m/z*: calc. for $C_{20}H_{20}N_2O_2$: 320.3842; found 320.208

Figure S6 MALDI-TOF-MS spectrum of 4-FMCSB

References:

1 M. J. Frisch, G. W. Trucks, H. B. Schlegel, G. E. Scuseria, M. A. Robb, J. R. Cheeseman, G. Scalmani, V. Barone, G. A. Petersson, H. Nakatsuji, X. Li, M. Caricato, A. V Marenich, J. Bloino, B. G. Janesko, R. Gomperts, B. Mennucci, H. P. Hratchian, J. V Ortiz, A. F. Izmaylov, J. L. Sonnenberg, D. Williams-Young, F. Ding, F. Lipparini, F. Egidi, J. Goings, B. Peng, A. Petrone, T. Henderson, D. Ranasinghe, V. G. Zakrzewski, J. Gao, N. Rega, G. Zheng, W. Liang, M. Hada, M. Ehara, K. Toyota, R. Fukuda, J. Hasegawa, M. Ishida, T. Nakajima, Y. Honda, O. Kitao, H. Nakai, T. Vreven, K. Throssell, J. A. Montgomery Jr., J. E. Peralta, F. Ogliaro, M. J. Bearpark, J. J. Heyd, E. N. Brothers, K. N. Kudin, V. N. Staroverov, T. A. Keith, R. Kobayashi, J. Normand, K. Raghavachari, A. P. Rendell, J. C. Burant, S. S. Iyengar, J. Tomasi, M. Cossi, J. M. Millam, M. Klene, C. Adamo, R. Cammi, J. W. Ochterski, R. L. Martin, K. Morokuma, O. Farkas, J. B. Foresman and D. J. Fox, 2016, preprint.

2 K.-T. Li, Y.-B. Lin and D.-Y. Yang, *Org Lett*, 2012, 14, 1190–1193.

3 M. Ghashghaee, B. Kaboudin, F. Kazemi, M. Hassan, H. Fukaya and H. Yanai, *J Org Chem*, 2024, 89, 7662–7670.
